# Modeling Retinitis Pigmentosa: Retinal Organoids Generated From the iPSCs of a Patient With the USH2A Mutation Show Early Developmental Abnormalities

**DOI:** 10.3389/fncel.2019.00361

**Published:** 2019-08-07

**Authors:** Yonglong Guo, Peiyuan Wang, Jacey Hongjie Ma, Zekai Cui, Quan Yu, Shiwei Liu, Yunxia Xue, Deliang Zhu, Jixing Cao, Zhijie Li, Shibo Tang, Jiansu Chen

**Affiliations:** ^1^Ophthalmology Department, The First Affiliated Hospital of Jinan University, Guangzhou, China; ^2^Key Laboratory for Regenerative Medicine of Ministry of Education, Jinan University, Guangzhou, China; ^3^Aier School of Ophthalmology, Central South University, Changsha, China; ^4^Shenzhen Aier Eye Hospital, Shenzhen, China; ^5^Aier Eye Institute, Changsha, China; ^6^Centric Laboratory, Medical College, Jinan University, Guangzhou, China; ^7^Institute of Ophthalmology, Medical College, Jinan University, Guangzhou, China

**Keywords:** retinitis pigmentosa, USH2A, iPSCs, organoid, RPE, basement membrane

## Abstract

Retinitis pigmentosa (RP) represents a group of inherited retinopathies with early-onset nyctalopia followed by progressive photoreceptor degeneration causing irreversible vision loss. Mutations in USH2A are the most common cause of non-syndromic RP. Here, we reprogrammed induced pluripotent stem cells (iPSCs) from a RP patient with a mutation in *USH2A* (c.8559-2A > G/c.9127_9129delTCC). Then, multilayer retinal organoids including neural retina (NR) and retinal pigment epithelium (RPE) were generated by three-step “induction-reversal culture.” The early retinal organoids derived from the RP patient with the USH2A mutation exhibited significant defects in terms of morphology, immunofluorescence staining and transcriptional profiling. To the best of our knowledge, the pathogenic mutation (c.9127_9129delTCC) in *USH2A* has not been reported previously among RP patients. Notably, the expression of laminin in the USH2A mutation organoids was significantly lower than in the iPSCs derived from healthy, age- and sex-matched controls during the retinal organogenesis. We also observed that abnormal retinal neuroepithelium differentiation and polarization caused defective retinal progenitor cell development and retinal layer formation, disordered organization of NRs in the presence of the USH2A mutation. Furthermore, the USH2A mutation bearing RPE cells presented abnormal morphology, lacking pigmented foci and showing an apoptotic trend and reduced expression of specific makers, such as MITF, *PEDF*, and RPE65. In addition, the USH2A mutation organoids had lower expression of cilium-associated (especially *CFAP43*, *PIFO*) and dopaminergic synapse-related genes (including *DLGAP1*, *GRIK1*, *SLC17A7*, and *SLC17A8*), while there was higher expression of neuron apoptotic process-related genes (especially *HIF1A*, *ADARB1*, and *CASP3*). This study may provide essential assistance in the molecular diagnosis and screening of RP. This work recapitulates the pathogenesis of USH2A using patient-specific organoids and demonstrated that alterations in USH2A function due to mutations may lead to cellular and molecular abnormalities.

## GRAPHICAL ABSTRACT

A model of USH2A mutation-associated 3D retinal developmental abnormalities.

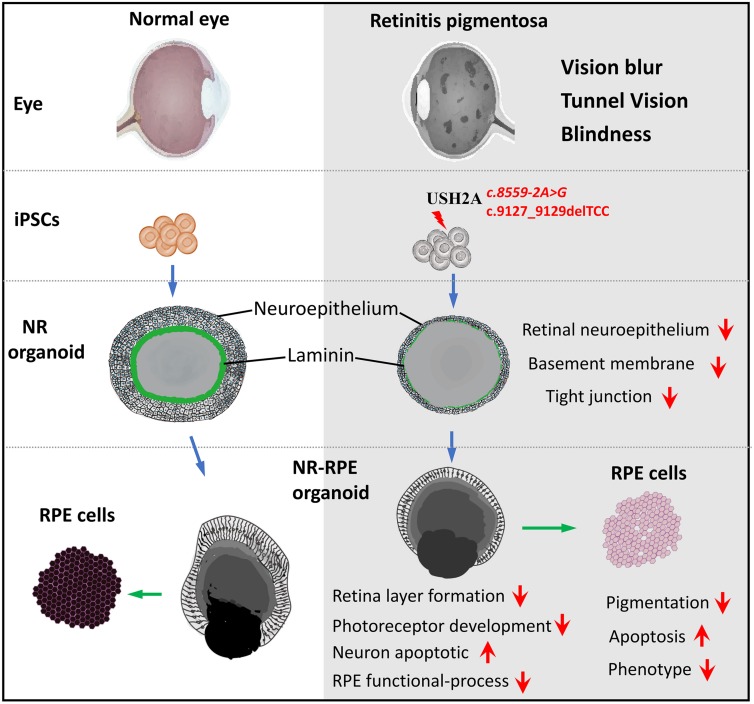

## Introduction

Retinitis pigmentosa (RP) is a group of inherited and progressive eye diseases that cause vision loss (Hartong et al., 2006). Unfortunately, RP disease is still incurable although RP patients have benefited from progress in research (Campochiaro and Mir, 2018; Dona et al., 2018). Initial RP symptoms include difficulty seeing at night and decreased peripheral vision due to rod- and cone-photoreceptor degeneration in the retinal structure (Shintani et al., 2009). As the disease progresses, the patient visual field (VF) gradually becomes only tunnel vision, eventually leading to dysfunction and blindness (Hartong et al., 2006). Most hereditary forms of RP are monogenic, and the inheritance patterns include autosomal dominant, autosomal recessive, X-linked, or maternal (mitochondria) (Rivolta et al., 2002). Currently, more than 95 RP pathogenic genes have been mapped and identified, among which 65 are implicated in the autosomal recessive form^1^. These known genes account for only approximately 60–70% of all RP cases and the other 40% have not been identified (Chen et al., 2018). Mutations of the *USH2A* gene in RP cases were discovered two decades ago (Weston et al., 2000). Over 600 different mutations in USH2A have been identified^2^ and mutations in this gene are by far the most frequent cause of autosomal recessive non-syndromic RP (12–25%) (Slijkerman et al., 2018).

The *USH2A* gene is located at chromosome 1q41 and contains 72 exons ranging in length from 127 bp to 78 kb, which encodes the protein USHERIN (van Wijk et al., 2004). The *USH2A* gene has two isoforms, A and B. Isoform A consists of 21 exons encoding ∼170 kDa of an extracellular protein (Weston et al., 2000), and isoform B is the full-length encoding an ∼580 kDa complex transmembrane protein (Liquori et al., 2016). USHERIN contains laminin EGF motifs, a pentraxin domain, a short intracellular region with a PDZ-binding motif, some fibronectin type III motifs and so on (Dona et al., 2018). It is generally considered that USHERIN is an important stabilizing component in the centrosome-cilium interface region of the photoreceptor, where it is fixed by interactions with HARMONIN, SANS and WHIRLIN (Chen et al., 2014; Sorusch et al., 2017). It has also been proposed that USHERIN might play an important role in vesicle trafficking between the inner and outer segments of the photoreceptor based on its protein interactions and localization (Reiners et al., 2006; Maerker et al., 2007). On the other hand, USHERIN may also play a key role in structural maintenance of the apical inner segment, the basal outer segment, and the connecting cilium (Insinna and Besharse, 2008; Nachury et al., 2010). Huang et al. demonstrated that photoreceptors synthesized the *USH2A* protein and selectively deposited it into the interphotoreceptor cell matrix (IPM). The USH2A protein also displays homology to laminin and other extracellular matrix (ECM) proteins containing laminin epidermal growth factor (LE) and fibronectin type III (F3) motifs. Therefore, USH2A could perform ECM functions, including providing an environment for mechanical and physiological support of the surrounding cells, attachment of cells to the underlying epithelium, and/or signals for differentiation (Huang et al., 2002).

Animal models are useful for understanding the biological function of the *USH2A* gene and the pathogenic mechanisms underlying USH2A’s role in RP. Liu et al. (2007) generated an *Ush2a* knockout mouse model in 2007. Their data showed that the ∼580 kDa long USHERIN isoform is the predominant form in photoreceptor cells and USHERIN holds the apical inner segment recess that wraps around the connecting cilia. They also found that more than one-half of the photoreceptors degenerated and the outer segments of photoreceptors became very short and disorganized in *Ush2a* knockout mice by 20 months. Recently, there have been two reports that found that defects or the absence of USHERIN produced early onset retinal dysfunction in zebrafish models, which were specifically represented by reductions in a-wave and b-wave amplitudes in zebrafish model larvae compared to wild-type larvae. Dona et al. (2018) showed that mutation of *ush2a* led to decreases in WHIRLIN and ADGRV1 levels at the periciliary region of the photoreceptor and increased the apoptosis of photoreceptors. Han et al. (2018) revealed that the photoreceptors progressively degenerated and rod degeneration occurred prior to cone degeneration in *ush2a* knockout zebrafish. The studies of these *USH2A* knockout or mutation animal models provide some clues to uncovering the function of the *USH2A* gene. However, solid evidence supporting the function of *USH2A* is still difficult to obtain for the human retina.

Induced pluripotent stem cells (iPSCs) are a favorable tool in modeling inherited retinal disease. Cells derived from skin, eye, blood, or urine of patients can be reprogrammed to become iPSCs and then differentiated into retinal cell types. A disease model is created by compared the phenotypes and genotype between diseased retinal cells and normal cells. The recent development of iPSCs has enabled researchers to recapitulate the retinal structure, physiology, functionality, pathological changes and mechanisms *in vitro* (Jin et al., 2011). Researchers have successfully revealed evidence of pathogenic mechanisms by using patient specific iPSCs. Megaw et al. (2017) showed that iPSCs-derived photoreceptors from *RPGR* mutation patients exhibited increased actin polymerization compared to the control, which was due to a disruption of cell signaling pathways regulating actin turnover. Schwarz et al. (2017) validated that iPSC-derived 3D optic cups from a patient with the *RP2* mutation (p. R120X) develop normally, but the photoreceptors in the optic cups displayed reduced Kif7 staining at their cilia tips compared with controls. Jin et al. (2012) revealed that diffuse distribution of RHO protein is associated with endoplasmic reticulum stress in an iPSCs disease model derived from a RP patient with a CHO mutation.

Remarkably, three-dimensional (3D) culture technology allows embryonic stem cells (ESCs)/iPSCs in dishes to utilize self-assembly characteristics to generate retinal organoids, which reflect major structural and functional properties of real organs. Organoid technology is therefore, conducive to increasing insight into human retinal development, providing new avenues for drug screening, and serving as disease models (Li and Izpisua Belmonte, 2019). Eiraku et al. (2011) generated dynamic optic cup structures from mouse ESCs by 3D culture reminiscent of retinal development *in vivo*. Then, they demonstrated that optic cup organoids can be produced by self-organization of human ESCs, and the optic cup organoids have the capacity to form multilayered neural retina (NR) containing both rods and cones (Nakano et al., 2012). There are some differences between human ESCs and mouse ESCs derived optic cup organoids in structure and morphogenetics. More recently, a method of optic cup organoids produced by iPSCs has also become feasible. Meyer et al. found that optic vesicle-like structures arose at the appropriate time from human iPSCs by 3D culture of retinogenesis, and the vesicle-like structures from iPSCs are indistinguishable from those derived from human ESCs (Meyer et al., 2011). Zhong et al. (2014) showed that human iPSCs enable the generation of 3D retinal cups that contain mature photoreceptors with outer-segment-disc and photosensitivity. Sharma et al. (2017) reported that iPSCs and iPSC-derived retinal organoids carrying the *TRNT1* mutation exhibited a deficit in autophagy and inefficient expression of full-length TRNT1 protein. Deng et al. (2018) also demonstrated that urine derived from a RP patient with a RPGR mutation could be reprogrammed into iPSCs, which enabled differentiation into RPE cells and retinal organoids with shorted cilium. Thus, this cutting-edge technology provides a possibility for understanding the effect of mutations in *USH2A* on the development of human retinal organogenesis.

In the present study, we identified a novel pathogenic mutation in the *USH2A* gene (c.8559-2A > G/c.9127_ 9129delTCC) that lead to non-syndrome RP. The iPSCs were generated from the urine cells (UCs) of a RP patient with a *USH2A* mutation, and 3D retinal organoids were generated and differentiated into retinal pigment epithelium (RPE) cells to recapitulate the disease *in vitro*. We compared the 3D retinal organoids formation of iPSCs with the USH2A mutation with those derived from age- and sex-matched normal iPSCs by integrating the morphology and phenotype of retinal differentiation data with transcriptome profiling. We were able to demonstrate abnormal developmental features of the USH2A mutation in 3D retina and RPE cells.

## Materials and Methods

### Clinical Diagnosis of RP

The proband and his family members were evaluated at Shenzhen Aier Eye Hospital (Shenzhen, China). The proband underwent a slit-lamp examination, visual acuity testing, fundus photography, spectral domain optical coherence tomography (SD-OCT), and full-field electrophysiological testing (ERG). The diagnosis of RP was established based on the appearance of abnormal pigmentation in fundus examination, and an extinguished aptitude on full-field rod ERG. Computerized testing of the VFs. The proband signed informed consent for participation in this study. The study was approved by the ethical committee of Aier Eye Institution and adhered to the tenets of the Declaration of Helsinki.

### The Isolation of Urine Cells From the RP Patient

Urine cells were collected from 400 mL of fresh urine that was centrifuged, then the pellet obtained was washed with PBS, resuspended in urine cell isolation medium (UCI; Cellapy Biotechnology, Beijing, China), and plated onto 12-well plates. Six to seven days after cell seeding, the medium was replaced with urine cell expansion medium (UCE; Cellapy Biotechnology).

### Generation of RPiPS Cells From UCs

Induced pluripotent stem cells were produced by the integration-free CytoTune-iPS 2.0 Sendai Reprogramming Kit as previously reported (Guo et al., 2018). Prior to viral transduction, 2 × 10^5^ UCs were seeded onto one well of a Matrigel-coated 12-well plate. After 24 h, the medium was discarded and we added fresh UCE medium containing 2.5 ng/ml of basic FGF2. After 72 h of transduction, the medium was discarded and we added a fresh 1:1 (v/v) mix of UCE (no FGF2) and E6 (10 ng/ml FGF2) media. At the fourth day post transduction, the medium was changed to E6 medium (10 ng/ml FGF2) until colonies appeared. At that time the medium was changed to E8 medium. It should be noted that the culture medium before and after cell passage was consistent in reprogramming induction. At least three patient-derived iPSCs clones were used for subsequent experiments.

### Generation of Retinal Organoid and RPE Cells

3D retinal organoids were generated from iPSCs using a method previously reported (Kuwahara et al., 2015). Briefly, iPSCs were digested into single cells and reseeded in low-cell-adhesion 96-well plates with V-bottomed conical wells at a density of 12,000 cells per well in NR induction medium supplemented with 20 μM Y-27632 under 5% CO_2_ conditions. On day 6, recombinant human BMP4 was added into a culture well at a concentration of 1.5 nM, and its concentration was diluted into half by half medium change every third day. On day 18, the NR containing aggregates were transferred onto low-cell-adhesion 6-well plates (6 aggregates per well) in RPE-induction medium supplement with CHI99021 (3 μM) and SU5402 (5 μM) under 5% CO_2_ conditions for 6 days culture. On day 24, the aggregates with RPE-like were cultured in NR and RPE induction medium under 40% O_2_, 5% CO_2_ conditions for 6 days culture for long term culture. RPE cells were differentiated from 3D retinal organoids following the method previously published with some modifications (Reichman et al., 2017). On day 34, identified pigmented patches were cut around from retinal organoid aggregate and cultured onto 6-well plates coated with 0.1% gelatin with RPE medium containing DMEM/F12, 1% MEM non-essential amino acids, 1% N2 supplement, 100 U ml-1 penicillin and 100 μg ml−1 streptomycin. The medium was changed every 2–3 day.

### RNA-Seq Analysis

The RNA-seq analysis was performed as previously described (Cui et al., 2018). Briefly, total RNA of organoids in RPiPSCs and NiPSCs was extracted. Each group had three repetitions. The realization of the RNASeq was subcontracted to Chi Biotech Co., Ltd. (Shenzhen, China). The RNA of samples was submitted for the library construction. After the library sequencing, RPKM (Reads Per Kilo bases per Million reads) was calculated to obtain normalized gene expression levels. FANSe2 was used to map the original RNA-seq to the reference transcriptome sequence. The correlation coefficients between gene expression levels were calculated and plotted as a correlation heatmap. The screening threshold for the differentially expressed genes (DEGs) was set to: |log2 (FoldChange)| > 1 and *P*-Value < 0.05. Gene ontology (GO) analysis was performed using TopGO software (version 2.18.0). Pathway enrichment analysis was primarily based on the Kyoto Encyclopedia of Genes and Genomes (KEGG) database. KOBAS software (kobas2.0-20150126) was used, and comparisons between the two groups were made using the hypergeometric test.

### QPCR

The qPCR protocol was performed as described in our previous report (Guo et al., 2017). Total RNA was extracted using a Tissue RNA Miniprep Kit. RNA (1 mg) was reverse transcribed into cDNA by using a ReverTra Ace qPCR RT Kit (TOYOBO, Japan). A qPCR reaction in a 20 μl total volume mixture containing 2 × SYBR, 250 nM of forward and reverse primers, and 10 ng of cDNA. The primers used are shown in Table 1.

**TABLE 1:** List of primers.

**Primers**		**Sequences (5′to 3′)**	**Size (bp)**	**GeneBank Accession Number**
*GAPDH*	Sense	GGTCGGAGTCAACGGATTTG	219	BC059110
*GAPDH*	Antisense	TGGAAGATGGTGATGGGATT		
PAX6	Sense	ACATCTGGCTCCATGTTGGG	184	NM_013435
PAX6	Antisense	ATAACTCCGCCCATTCACCG		
*RAX*	Sense	CTCCTCTCCGTCTCCAAAGC	275	NM_003106.
*RAX*	Antisense	TCCCGTCGTCCTTGGTAAAC		
*SIX6*	Sense	GGTTCAAAAACCGCCGACAA	165	NM_007374
*SIX6*	Antisense	CCTTGCTGGATAGACTGGCG		
*CHX10*	Sense	TTCAACGAAGCCCACTACCC	167	NM_182894
*CHX10*	Antisense	TAGAGCCCATACTCCGCCAT		
*CASP3*	Sense	TTGGAACCAAAGATCATACATGGAA	178	NM_004346
*CASP3*	Antisense	TGAGGTTTGCTGCATCGACA		
*MSH2*	Sense	GACTTCTATACGGCGCACGG	105	NM_000251
*MSH2*	Antisense	CAGATTCTTTGCTCCTGCCG		
*ADARB1*	Sense	GGCATGGAGAGCTTAAGGGG	179	NM_015833
*ADARB1*	Antisense	CTTGACTGGCGGAGACTGTT		
*HIF1A*	Sense	GCCAGACGATCATGCAGCTA	171	NM_001530
*HIF1A*	Antisense	CTGGTCAGCTGTGGTAATCCA		
*PEDF*	Sense	AGTGTGCAGGCTTAGAGGGAC	107	NM_002615
*PEDF*	Antisense	CCCGAGGAGGGCTCCAATG		
*RPE65*	Sense	CCACCTGTTTGATGGGCAAG	162	NM_000329
*RPE65*	Antisense	CAGGGATCTGGGAAAGCACA		
*TJP1*	Sense	AGCCATTCCCGAAGGAGTTG	175	NM_003257
*TJP1*	Antisense	ATCACAGTGTGGTAAGCGCA		
*MITF*	Sense	AGCTTGTATCTCAGTTCCGC	200	NM_198177
*MITF*	Antisense	ATGGCTGGTGTCTGACTCAC		
*OTX2*	Sense	CCTCACTCGCCACATCTACT	196	NM_021728
*OTX2*	Antisense	GTTTGGAGGTGCAAAGTCGG		
*COL4A6*	Sense	ATTTTGGTCGGTGCTCCCTG	166	NM_001847.3
*COL4A6*	Antisense	CTCGGTCAGGCACAACGTAA		
*LAMb1*	Sense	TTCAGTTTCTTAGCCCTGTGC	175	NM_002291.3
*LAMb1*	Antisense	CGATACAGTAGGGTTCGGGC		
*TNC*	Sense	CCAAAACATTTCTGGACAGTACCT	182	NM_002160.4
*TNC*	Antisense	CCCCTCCCACTTGACCACTA		
*CLDN4*	Sense	AGCCTTCCAGGTCCTCAACT	167	NM_001305.4
*CLDN4*	Antisense	GCGAGGTGACAATGTTGCTG		
*CLDN19*	Sense	AACCCAAGCACACCTGTCAA	189	NM_148960.2
*CLDN19*	Antisense	AACAACTGGTTCTCGGGCAG		

**TABLE 2:** List of antibodies.

**Antigen**	**Host species**	**Company or provider**	**Cat. no.**
OCT4	Rb	Proteintech	11263-1-AP
SSEA4	MS	Cell Signaling	4755s
TRA-160	MS	Abcam	ab16288
SOX2	Rb	Proteintech	11064
AFP	Rb	Abclonal Technology	A0200
SMA	Rb	ThermoFish	PA5-16697
Nestin	Ms	Santa Cruz Biotechnology	SC23927
RAX	Ms	Antibodies	AA 104-206
N-cadherin	Ms	R&D	MAB13881
CDH2	Sheep	R&D	AF6426
CHX10	Ms	Santa	sc-365519
PAX6	Rb	Abcam	ab154253
aPKC	Ms	Abcam	ab11723
Ki67	Rb	Abcam	ab15580
RPE65	Ms	Abcam	ab13826
TYR	Rb	Abcam	ab180753
MITF	Rb	Abcam	ab122982
ZO-1	Ms	ThermoFish	Cat # 33-9100
Laminin	Rb	Abcam	ab11575

### Immunofluorescence Staining

The immunofluorescence staining was performed as described in our previous report (Guo et al., 2015). Samples were fixed in paraformaldehyde and permeabilized with 0.1% Triton X-100 in PBS. The samples were incubated with the primary antibody overnight and then incubated with the secondary antibodies. Finally, the samples were incubated with DAPI for nuclear staining. The antibodies used are shown in Table 2.

### Data Analysis

All experiments were performed with at least 3 different iPSCs clones from patient and healthy control, 6–8 retinal organoids were required in each experiment. All data are presented as the averages ± standard deviations from 3–6 independent experiments (3–6 iPSCs clones). Statistical analyses were conducted using a two-sided unpaired Student’s *t*-test to compare differences between two groups. *p* < 0.05 was considered statistically significant.

## Results

### Identification of a Novel Pathogenic Mutation of the *USH2A* Gene in RP

**FIGURE 1:**
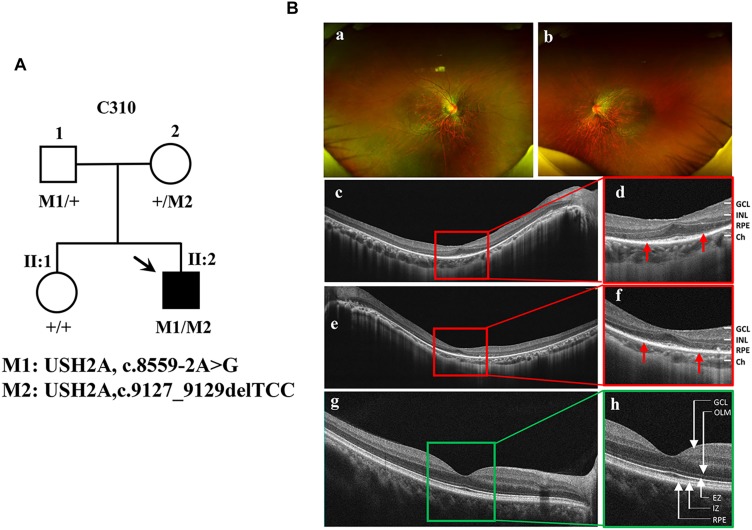
Identification of a novel pathogenic mutation of the USH2A gene in RP. **(A)** Pedigrees and mutations segregating in family C310. Males and females are represented by squares and circles, respectively. The symbols for affected family member are filled. The symbols for proband with black arrow. The genotype of each evaluated individual is shown below the individuals’ symbol and identification number. Abbreviations: Wild type (+); c.8559-2A > G (M1); c.9127_9129delTCC (M2). **(B)** Fundus images of the proband and health control. Fundus photographs showed the abnormal pigmentation in the peripheral area in both eye of the proband **(a,b)**. Loss of outer limiting membrane (OLM), ellipsoid zone (EZ) and interdigitation zone (IZ) was visualized by SD-OCT in the proband **(c–f)**, when comparing to the normal eye **(g,h)**.

The proband (Figure 1A, arrow) is a man who was 27 years old at the time of diagnosis. The patient exhibited typical clinical features of RP, including initial nyctalopia and visual acuity impairment. Best corrective visual acuity (decimal) of the proband was 20/25 (0.8, feet equivalent) in his both eyes. There was VF constriction and patient left eye presented more severe VF loss. His clinical phenotype in both eyes showed an extensive leopard spot-like pattern with a granular appearance that had individual pigment mottling under fundus images (Figure 1B).

SD-OCT images revealed that the outer limiting membrane, ellipsoidal zone and interdigitation zone had disappeared from almost all of the retina, except for a residual in the fovea. The thickness of the retinal neuroepithelial and outer nuclear layers (ONLs) was significantly decreased in both eyes (Figure 1B). The ERG results showed that decreased or distinguished b wave amplitude in scotopic ERG, which suggested the loss of rod function. The cone function was also impaired in photopic ERG testing (Supplementary Figure S1). His vestibular and hearing function were normal. Genomic sequencing data confirmed that the patient carries two compound heterozygous mutants in the *USH2A* gene. One was c.8559-2A > G inherited from the patient’ father, which has been reported to be pathogenic when in combination with a missense mutation c.11806A > C (p.T3936P) (Dai et al., 2008). The other was c.9127_9129delTCC inherited from the patient’ mother, which is a novel deletion mutation in *USH2A* (Supplementary Figure S2). However, the patients’ parents had no disease phenotypes despite carrying these mutations. Taken together, the patient was diagnosed as the proband with non-syndrome RP disease by clinical phenotype and genotype demonstration.

### Reprogramming Patient-Specific Urine Cells

**FIGURE 2:**
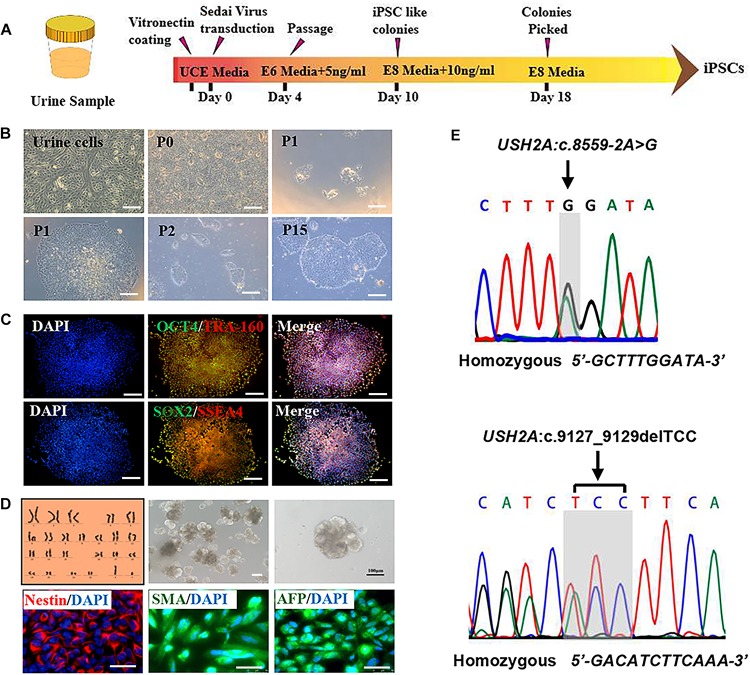
Characteristics of iPSCs derived from RP patient urine cells. **(A)** A schematic of RP patient-specific iPSCs generated by use of an integration-free Sendai Reprogramming Kit. **(B)** Morphology of RP patient-specific urine cells **(a)** and iPSCs in different passages **(b–f)**. **(C)** Immunofluorescence staining analysis results of the pluripotency markers OCT4, TRA-160, SOX2, and SSEA4 in RP patient-specific iPSCs. **(D)** Normal karyotype (46, XY), embryoid body formation, and spontaneous differentiation experiments show that the RP patient-specific iPSCs had the capacity to form three germ layers, which was verified by immunofluorescence staining with Nestin, SMA, and AFP. **(E)** The USH2A mutation was confirmed in RP patient-specific iPSCs. (scale bar = 100 μm).

RP patient-specific and control (age- and sex- matched healthy donor) UCs were harvested from donated urine samples and reprogrammed to induced pluripotent stem cell (iPS) lines by using an integration-free CytoTune^TM^-iPS 2.0 Sendai Reprogramming Kit (Figure 2A). The morphology of the RPiPSCs clones resembled that of typical iPS clones (Figure 2B). Immunofluorescence staining showed that RPiPSCs at passage 7 positively expressed pluripotency markers Oct4, Sox2, SSEA4, and TRA-160 (Figure 2C). Immunofluorescence staining also revealed that the RPiPSCs had the ability to form embryoid bodies and spontaneous differentiated into three germ layer cells, which were positive for Nestin, SMA, and AFP (Figure 2D). The RPiPSCs clones showed a normal karyotype (46, XY) and the USH2A mutations were confirmed (Figures 2D,E).

### Association of the USH2A Mutations With Abnormal Organoid Induction and Increased Apoptosis

**FIGURE 3:**
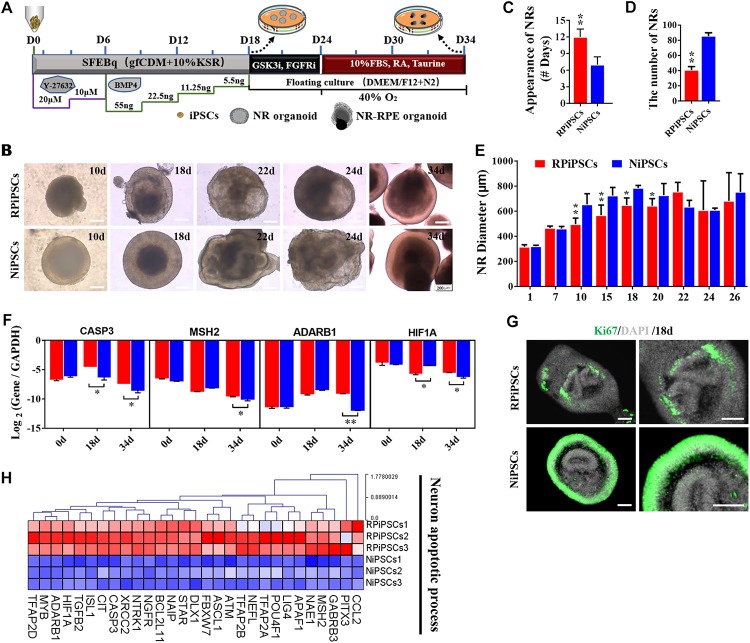
USH2A mutation neural retina (NR) organoids have abnormal organoid induction and increased apoptosis. **(A)** A schematic representation of patient-specific iPSC differentiation along the NR organoid lineage. Brightfield images of developing NR over time **(B)** and USH2A mutation iPSCs generated NR at a slower pace **(C)**, less efficiently **(D)**, and with a smaller diameter **(E)** versus controls. **(F)** qPCR analysis revealed mRNA levels of neuron apoptosis cell specific markers *CASP3*, *MSH2*, *ADARB1*, and *HIF1A* (*GAPDH* gene as a control). **(G)** Immunostaining of retinal organoids showing the expression of cell-proliferation marker Ki67. **(H)** Neuron apoptotic process related DEGs at day 34 were normalized by *Z*-scores. Red represents upregulated expression. Blue represents downregulated expression, Bar is represents *Z*-score. (*n* = 3 independent experiments; each experiment need 2–4 organoids; scale bar = 200 μm).

To establish the disease model of RP with mutated USH2A *in vitro*, we generated NR – RPE conversion organoids from RPiPSCs and normal iPSCs (Figure 3A). The organoids displayed NR and RPE features after 34 days of NR-RPE conversion culture in the RP and normal group (Figure 3B). Temporal analysis revealed a delay (approximately 4–6 days) in the self-forming of NRs for USH2A mutation iPSCs compared to controls (Figure 3C). Quantification data showed that fewer NR organoids were induced from the RPiPSCs than those in the normal group (Figure 3D). The organoid diameters among those with the USH2A mutation were smaller than the controls at days 8–20 (Figure 3E). Additionally, the most peripheral cells of the organoids were more likely to fallen off from the USH2A mutation organoids during the cultivation (Supplementary Figure S3A). Furthermore, qPCR analysis revealed that the expression levels of apoptosis related genes, such as CASP3, MSH2, ADARB1, and HIF1A, were significantly increased in the USH2A mutation group at days 34 compared to the control (Figure 3F). According to the results of DEGs and GO enrichment of RNA-Seq analysis, the neuronal apoptosis-related genes were significantly upregulated in the RP groups compared with the control group at day 34 (Figure 3H). Additionally, a comparison of cell proliferation revealed fewer Ki67 positive cells in the USH2A mutation versus the control group (Figure 3G and Supplementary Figure S3B). These results collectively suggest that the USH2A mutation in iPSCs is associated with abnormal NR organoids induction, which is accompanied by decreased proliferation.

### Association of the USH2A Mutation With Defective Retinal Neuroepithelial and Photoreceptor Development

**FIGURE 4:**
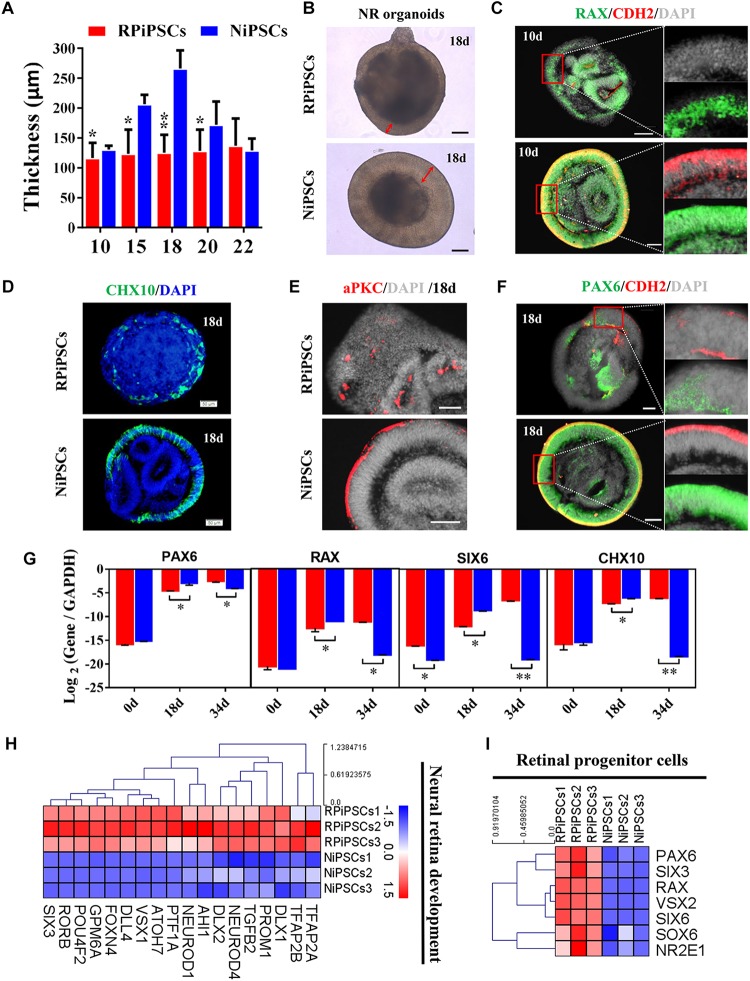
USH2A mutation iPSCs generate retinal organoids with defective retinal neuroepithelial and RPC development. **(A)** The thickness of retinal neuroepithelium in RP-organoids at days 15–22. **(B)** Brightfield images of developing retinal neuroepithelium at day 18. **(C)** Immunofluorescence staining shows the cells in retinal organoids co-expressing RAX and CDH2 (N-cadherin) immunoreactivity at day 10. **(D)** Immunofluorescence images shows the cells in NR organoids expressing CHX10 immunoreactivity at day 18. **(E,F)** Immunostaining of NR organoids showing the expression of specific markers PAX6, N-cadherin, and aPKC at day 18. **(G)** qPCR analysis reveals mRNA levels of RPCs specific markers *PAX6*, *RAX*, *SIX6*, and *CHX10* (*GAPDH* gene as a control). **(H,I)** Neural retina development and RPCs related DEGs at day 34 were normalized by *Z*-scores. Red represents upregulated expression. Blue represents downregulated expression, Bar is represents *Z*-score. (*n* = 3 independent experiments; each experiment need 2–4 organoids; scale bar = 200 μm).

The thickness of the retinal neuroepithelium in the RP-organoids was significantly reduced compared with the control group at days 15–22 (Figures 4A,B). To determine whether there was an adverse effect of the USH2A mutation on retinal organogenesis and neuroepithelium, we examined the expression of factors that regulate eye development growth (RAX, PAX6 and CHX10) and polarized guidance (N-cadherin and aPKC). Coimmunostaining analysis of the organoids presented a significantly reduced proportion of RAX^+^ and N-cadherin^+^ cells in the USH2A mutation group than in the controls at day 10 (Figure 4C and Supplementary Figure S4A). Over time, we observed that significantly fewer CHX10^+^, PAX6^+^, N-cadherin^+^, and aPKC^+^ cells expressed immunoreactivity corresponding to these factors in the USH2A mutation groups compared with controls at day 18 (Figures 4D–F and Supplementary Figures S4B–E). qPCR analysis also showed that the mRNA expression level of the RPCs genes, PAX6, RAX, SIX6, and CHX10, were significantly lower in the USH2A mutation group than the control at day 18 (Figure 4G). However, at day 34, the mRNA expression levels of these genes were expressed higher in the USH2A mutation group than the control group, which is consistent with the significantly upregulated expression of retinal development related- and RPCs related-genes detected by RNA-seq analysis (Figures 4G–I).

**FIGURE 5:**
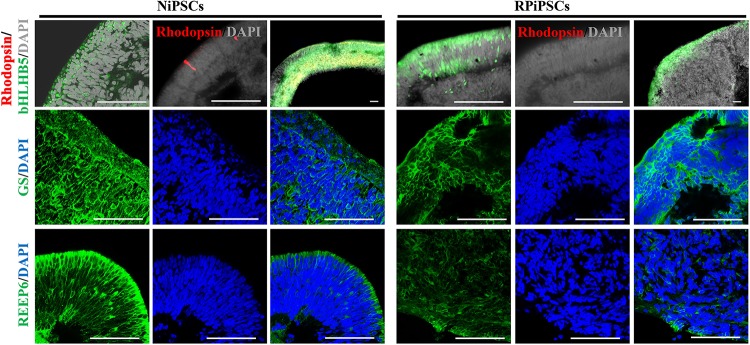
Differentiation of neural retinas in organoids at day 86. bHLHB5, glutamine synthetase (GS) and Reep6 are markers of amacrine cells, Müller cells, and rod photoreceptors, respectively. Rhodopsin immunoreactivity is most intense in the rudimentary outer segments. The stratified neural retinas differentiated from organoids were disordered organization and negative for Rhodopsin in USH2A mutation group, while the normal organoids differentiated retinal cells, such as photoreceptors, Müller cells, and Amacrine cells all arranged in their proper layers and weak expressed Rhodopsin. The bHLHB5, GS, Reep6 were positively expressed in the RP and normal organoids. Nuclei were stained with DAPI (blue). (*n* = 2 independent experiments; each experiment need 2 organoids; scale bar = 100 μm).

Moreover, to further understand the effect of USH2A mutation on neuroepithelial develop into a stratified NR tissue, we analyzed the expression of Reep6 (photoreceptors), GS (Müller glia), bHLHB5 (amacrine cells) by immunofluorescence. After 86 days of retina differentiated culture, the NR-RPE organoids displayed NR features and positive for bHLHB5, GS, Reep6 staining in the RP and normal group. However, the stratified NR tissue contain major retinal cell types were disordered organization in USH2A mutation group, while the normal organoids differentiated retinal cells, such as photoreceptors, Müller cells, and Amacrine cells all arranged in their proper layers (Figure 5). Meanwhile, there was a significantly decreased proportion of bHLHB5^+^, GS^+^, and Reep6^+^ cells in the USH2A mutation groups compared with controls. Furthermore, at day 86 Rhodopsin immunoreactivity showed negatively expression in the USH2A mutation organoids and instead revealed weak expression at the apical surface where developing photoreceptors were located in normal organoids (Figure 5). These results demonstrate that the USH2A mutation in iPSCs is associated with aberrant organoids polarization, defective neuroepithelium, and abnormal RPCs and photoceptors differentiation.

### Association of USH2A Mutations With Abnormal RPE Development and Growth

**FIGURE 6:**
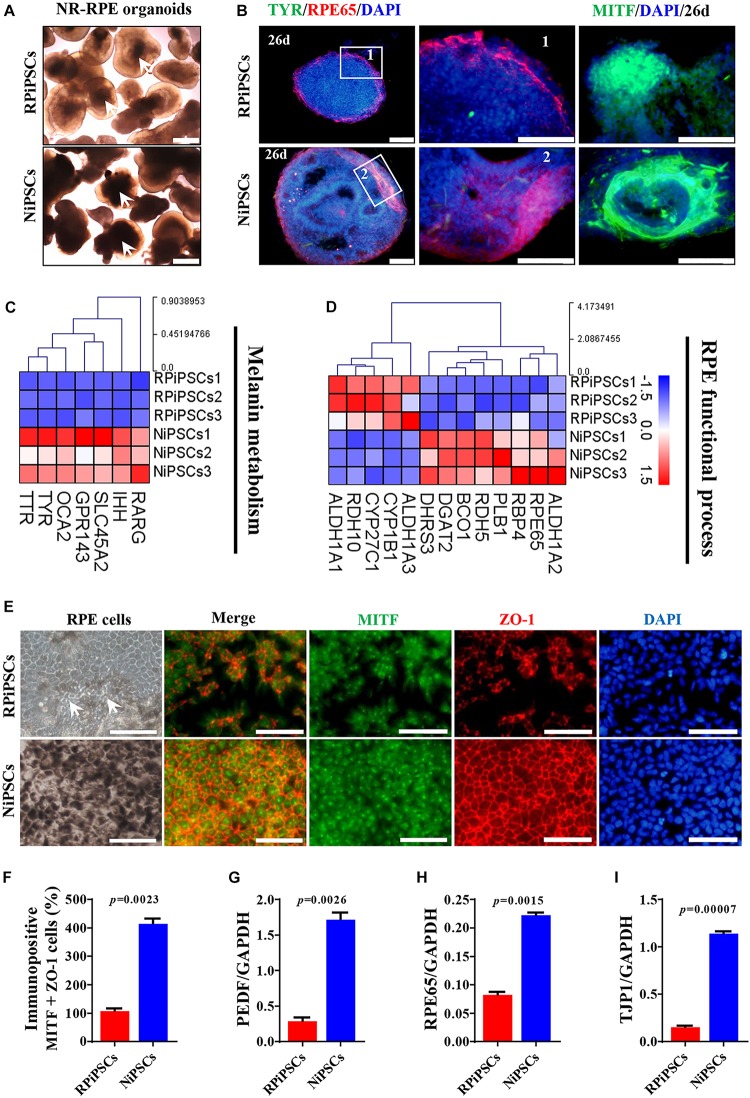
USH2A mutation iPSCs generate abnormal RPE development and growth. **(A)** Brightfield images of developing NR-RPE organoids at day 34. **(B)** Immunostaining of NR-RPE organoids showing the expression of RPE cell specific markers TYR, RPE65, and MITF. **(C,D)** Melanin metabolism and RPE functional process related DEGs at day 34 were picked and normalized by Z-scores. Red represents upregulated expression, blue represents downregulated expression, Bar is represents *Z*-score. **(E)** Immunostaining of RPE cells derived from NR-RPE organoids showing the expression of RPE cell specific markers MITF and ZO-1. **(F)** A separate bar graph shows the percentage of MIFT^+^ ZO-1^+^ cells for immunostaining in a merged image. **(G–I)** qPCR analysis revealed mRNA levels of RPE cell specific markers *PEDF*, *RPE65*, and *TJP1* (*GAPDH* gene as a control). (*n* = 3 independent experiments; each experiment need 2–4 organoids. Scar bar = 100 μm).

To better understand the effect of the USH2A mutation on RPE cells differentiated from RPiPSCs, we detected the morphology, phenotype and transcriptome of RPE. After NR-RPE conversion culture, the NR-RPE organoids showed RPE features as distinct pigmented foci in the RP and normal group at day 34. However, the pigmented foci in the RP organoids were significantly less than those in the control (Figure 6A). The immunofluorescence staining data showed the NR-RPE organoids were positive for RPE cell markers RPE65 and MITF in both groups at day 26. However, there was a significantly decreased proportion of RPE 65^+^ and MITF^+^ cells in the USH2A mutation groups compared with controls (Figure 6B and Supplementary Figures S5A,B). The qPCR analysis showed that the mRNA expression levels of OTX2 and MITF were lower in USH2A mutation organoids than those in the control at days 18–34 (Supplementary Figure S5C). Furthermore, RNA-seq data at day 34 showed that the major upregulated genes involved in the RPE functional process included ALDH1A3 and RDH10, while the major downregulated genes were RPE65, RDH5, and DHRS3 (Figure 6C). Melanin metabolism-related genes were all downregulated, especially the markers TYR, OCA2, RARG, and GPR143 at day 34 (Figure 6D).

Moreover, the adherent RPE cells at passage 2 differentiated from control organoids expressed many more RPE features than those in the USH2A mutation organoids. We observed abnormal morphology and absent melanin in RPE cells derived from the USH2A mutation organoids, while the RPE cells in the control presented a classic pigmented, cobblestone morphology (Figure 6E). Immunofluorescence staining assay revealed that there were significantly fewer MITF and ZO-1 positive cells in the USH2A mutation group compared to the control (Figures 6E,F). qPCR analysis also revealed that the RPE cells in the USH2A mutation group expressed lower levels of transcripts corresponding to RPE65, TJP1, and a mature marker, PEDF, compared to control (Figures 6G–I). Additionally, the RPE cells differentiated from USH2A mutation organoids displayed atrophic trends after passage in culture. Together, these observations demonstrate that the USH2A mutation is associated with abnormal RPE development and phenotype expression as well as increased cell death in *de novo* generated RPE cells.

### Association of the USH2A Mutation With Aberrant Basement Membrane and Tight Junctions

**FIGURE 7:**
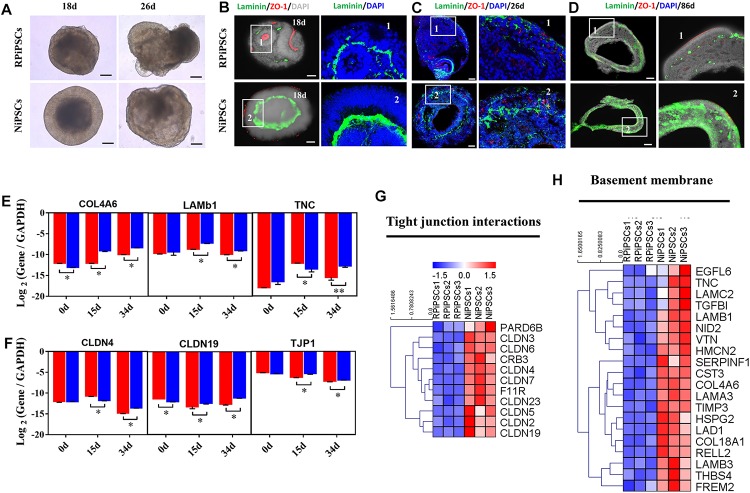
USH2A mutation iPSCs generated retinal organoids have aberrant basement membrane and tight junctions. **(A)** Brightfield images of developing retinal organoids at day 18 and 26. **(B–D)** Immunostaining of retinal organoids showing the expression of Laminin and ZO-1 at day 18, 26, and 86. **(E,F)** qPCR analysis reveals mRNA levels of basement membrane and tight junction regulators, *COL4A6*, *LAMb1*, *TNC*, *CLDN4*, *CLDN19*, and *TJP1* (*GAPDH* gene as a control). **(G,H)** Basement membrane and tight junction interactions associated DEGs at day 34 were picked and normalized by *Z*-scores. Red represents upregulated expression, blue represents downregulated expression, Bar is represents *Z*-score. (*n* = 5 independent experiments; each experiment need 4–6 organoids; scale bar = 200 μm).

We found that approximately 40% of the organoids in the USH2A mutation group degraded at day 19 of organoid induction. The most peripheral cells of the organoids began to shed and underwent apoptosis by day 26 (Figure 7A). Notably, fluorescent staining showed that the expression of the basement membrane marker Laminin in the USH2A mutation group was significantly lower than that in the control group at day 18 (Figure 7B and Supplementary Figure S6A). The Laminin^+^ expression was also lower in the USH2A mutation group than that in the control at day 26 and day 86 (Figures 7C,D and Supplementary Figure S6B). Likewise, the qPCR results showed that basement membrane markers COL4A6, LAMb1, and TNC were downregulated at day 15 and day 34 in the USH2A mutation group compared to the control organoids. Moreover, we also found that the tight junction interactions related transcription factors, such as CLDN4, CLDN19, and TJP1, were also significantly downregulated at day 15 and day 34 in the USH2A mutation group compared to the control organoids (Figures 7E,F). Furthermore, the RNA-seq data showed that the expression levels of basement membrane-associated mRNAs in the USH2A mutation group were significantly lower than those in the control group, such as COL4A6, CST3, and LAMA3 (Figure 7H). Meanwhile, it was also found that the tight junction interactions related transcription factors also showed significant downregulation, such as 4, 3, and 7 of the CLDN family (Figure 7G). Together, these observations suggest that the USH2A mutation is associated with degeneration of the basement membrane, tight junction and other intercellular conjunctions.

### Association of the USH2A Mutation With Abnormal Gene Expression and Pathways by RNA-Seq Analysis

**FIGURE 8:**
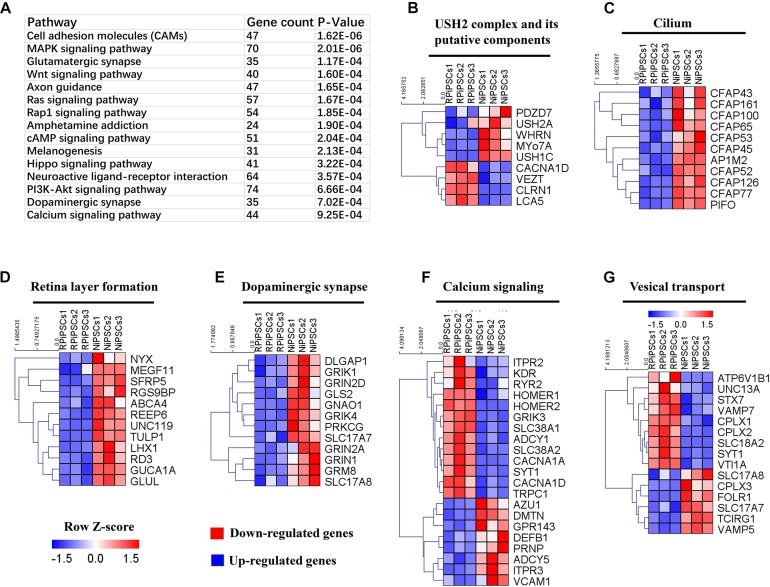
KEGG pathway and heatmaps of other major types of genes. **(A)** GO enrichment for KEGG pathways of the differentially expressed genes. USH2A complex **(B)**, cilium **(C)**, retina layer formation **(D)**, dopaminergic synapse **(E)**, calcium signaling **(F)**, and vesical transport **(G)** related DEGs were picked and normalized by *Z*-scores. Red represents upregulated expression. Blue represents upregulated expression. Bar is represents *Z*-score.

To understand the mechanism underlying the USH2A mutation affected genes and pathways, we examined RNA-seq expression of both groups’ organoids at day 34. Transcriptome data revealed significant differences between the control and RP organoids (Supplementary Figures S7A–C). There were 1853 DEGs upregulated and 1808 DEGs were downregulated (Supplementary Figures S7D). Pathway enrichment analysis showed that the USH2A mutation primarily affected the cell adhesion molecules pathway, followed by the MAPK signaling pathway, glutamatergic synapse, the Wnt signaling pathway and to a lesser extent, the calcium signaling pathway (Figure 8A and Supplementary Figure S7G). Remarkably, the differentially expressed gene ontology (GO) terms were those involved in the USH2 complex and its components, such as downregulated PDZD7, WHRN, MYO7A, and USH1C, and upregulated VEZT, CLRN1 and LCA5 (Figure 8B). qPCR analysis confirmed that the gene expression level of CLRN1 was abnormal in the USH2A mutation group compared to the control (Supplementary Figure S7E). Additionally, the cilium related genes were all downregulated, especially CFAP126, PIFO, and CFAP161 (Figure 8C). We also observed that “calcium signaling,” “retinal layer formation,” “dopaminergic synapse,” and “vesical transport” were among the top-ranking categories identified at the NR-RPE stage by GO analysis (Figures 8D–G). Together, the trend in these DEGs indicate that the USH2A mutation may adversely affect cell adhesion molecules, the glutamatergic synapse pathway and cilium, calcium signaling, dopaminergic synapse, and vesical transport related gene ontology terms in the early organoids.

## Discussion

Retinitis pigmentosa patients first gradually lose night vision and side vision, and then experience late central vision loss because of progressive loss of rod and cone photoreceptor cells (National Eye Institute, 2004). Mutations of the *USH2A* gene are the most common cause of RP and are found in around 10–15% of recessive RP and 30–40% of Usher syndrome type 2 cases (Sun et al., 2016; Huang et al., 2018). Here, the modeling RP retinal organoid was studied. We generated retinal organoids from the iPSCs of a patient with the USH2A mutation of c.8559-2A > G and c.9127_9129delTCC. The c.9127_9129delTCC was inherited from the patient’s mother, which is a novel deletion mutation in *USH2A* and may change the amino acid sequence and affect the protein features as predicted by Mutation Taster^3^. c.8559-2A > G was inherited from the patient’ father, which has previously been reported to be pathogenic when in combination with the missense mutation c.11806A > C (p.T3936P) (Dai et al., 2008). Sun et al. found that mutation c.8559-2A > G was the most frequent point mutation, which was only detected in Chinese and Japanese patients, accounting for approximately 19.1% of the identified USH2A mutations in 67 probands (Sun et al., 2018).

In this study, the proband is 27 years old at the time of diagnosis. This patient exhibited obviously visual acuity impairment, seriously decreased retinal neuroepithelial layers of SD-OCT, and severely abnormal ERG testing. From these clinical phenotype, we can reason that the patient has suffered from RP disease for quite a while and at an advanced stage of the pathology. On the other hand, we demonstrated for the first time that this RP patient iPSCs were able to form NR organoids but revealed abnormal early developmental features, including delayed self-organization, thinned retinal neuroepithelium, and disordered organization of retinal cells within a specific time window. Our results also revealed that the expression levels of neuron apoptosis related genes were up regulated in the USH2A mutation group compared to the control. These defection features in USH2A mutation organoid are corresponds to the disease characteristics of the RP patient.

USH2A-associated retinal degeneration in humans has a slow and progressive pathology. However, USH2A mutant retinal degeneration might also undergo some early pathological changes. Dona et al reported that zebrafish ush2a mutant’s models presented with early-onset retinal dysfunction, as evidenced by significantly reduced ERG a- and b-wave responses recorded at 5 days post fertilization (Dona et al., 2018). Ush2a knockdown experiments in zebrafish produced moderate levels of photoreceptor cell death in larvae. This cell death was restricted to photoreceptors and the retinas were morphologically normal (Aller et al., 2013). Photoreceptor degeneration in the Ush2a-/- mice is slowly progressive, similar to RP in human patients. However, GFAP was found to be up-regulated from as early as 2 months of age and remained so at the older ages in the Ush2a-/- mice. GFAP up-regulation is a non-specific indicator of photoreceptor degeneration and typically precedes overt cell loss (Liu et al., 2007). Our study also confirmed that retinal organoids generated from a RP patient with the USH2A mutation did exhibit early retinal developmental abnormalities.

According to the procedures of “induction-reversal” organoid culture in our study, firstly, the NR containing aggregates were cultured in NR induction medium for 18 days. Then, they were transferred to culture plates in RPE-induction medium from day 18 to day 24. Finally, they became NR-RPE like tissue in NR and RPE induction medium after day 24. Interestingly, HIF1A as well as RPCs related genes, such as PAX6, RAX, SIX6, and CHX10, at D18 in NR induction significantly higher expressed in controls and then at D34 in USH2A mutant organoids. Such changes in HIF1A may be related to the different oxygen concentration during the organoid culture. The oxygen concentration was 20% before 18 days, while we used 40% oxygen concentration during later culture. Additionally, corresponding changes in RPCs related markers might involve compensation growth and regeneration mechanism. McGuigan et al. (2017) reported that autosomal recessive RP with EYS Mutations showed decreased retinal and ONL as well as apparently increased inner nuclear layer (INL). Such observations of thickening INL coupled with thinning ONL have been noted in other inherited retinal degenerations (IRDs). The explanation has not been provided whereas this is only one of many exceedingly complicated and continuous retinal changes after photoreceptor loss, it could be a marker for a stage of remodeling (McGuigan et al., 2017). It was reported that acute damage to the retina triggers the reprogramming of Müller glial cells into retinal progenitor cells that are able to differentiate into all major types of retinal neurons including photoreceptors (Wan and Goldman, 2016). Dona et al uncovered that mutant ush2a zebrafish models showed no progressive loss of retinal photoreceptor cells under normal light conditions, although increased levels of photoreceptor apoptosis and impaired visual function were observed within the first week of life when larvae were challenged by exposure to constant illumination with 3000 lux of white light (Dona et al., 2018). The observed lack of progressive retinal degeneration in these mutants suggests that the rate of photoreceptor apoptosis might be compensated by the rate of photoreceptor regeneration when fish are raised in low intensity light. Furthermore, after RPE-induction, the pigmented domain of RPE is gradually increasing according to the procedures of “induction-reversal” organoid culture in our study. And NR-RPE like tissue exhibited a large semispherical domain of continuous NR epithelium with a small pigmented domain of thin and winding RPE in an adjacent location (Kuwahara et al., 2015). We suppose that the development of RPE cells helps the growth and differentiation of RPC, which is manifested by the increase in the thickness of neuroepithelial. The RPE cells are responsible for producing some of the molecular signals that influence RPC differentiation and for providing metabolic support for the photoreceptors in the mature retina (Lu and Barnstable, 2019). Zhu et al. (2011) showed that the hESCs derived RPE cells secrete a high level of PEDF, which can promote RPC proliferation and survival. But in first NR induction medium before day 18 (including day 20 just beginning RPE growth), USH2A mutations indeed induced defective and reduced thickness of the neuroepithelium. Therefore, in our study, RPCs in USH2A mutant organoids firstly underwent apoptosis and decreased expression, which triggers later remodeling and compensatory increased expression and by regenerative pathway and further intensified with RPE action.

In addition, a significantly decreased expression of the basement membrane markers Laminin, *COL4A6*, *LAMB1*, *TNC*, *LAMA3*, and *LAMC2* were confirmed by immunofluorescence staining, qPCR and RNA-Seq, which were connected with abnormal retinal organoids with USH2A mutant in this study. Firstly, decreased cell growth and increased cell apoptosis happen when there is improper basement membrane. We showed that there was a significantly smaller proportion of Ki67^+^ cells and more apoptosis in the USH2A mutant organoids, as well as upregulated neuro apoptotic process related genes including *CASP3*, *MSH2*, *ADARB1*, and *HIF1A.* Our results are broadly consistent with other perspectives. Urbano et al. (2009) demonstrated that mutation of the *Lamb1* gene not only prevented basement membrane from forming, but it also generated abnormal organogenesis with defective adhesion and cell migration in Drosophila. Laperle et al. (2015) found that knockdown of the *LAMA5* gene significantly increased human ESCs and iPSCs apoptosis but did not affect their pluripotency. Secondly, abnormal cell polarization can be affected in a situation with an abnormal basement membrane. we also demonstrated that the USH2A mutation organoids displayed sluggish self-assembly and chaotic polarization, which is reflected in their dramatic decrease in the expression level of aPKC, CDH2, and ZO-1 compared to normal organoids. Similarly, Huang et al. (2003) showed that laminin is essential for the formation of cell-anchored polymers, which are required for basement membrane assembly and epithelial cell polarization. Ivanovitch et al. (2013) revealed that the absence of *laminin1* caused misoriented AB polarity and pseudostratified neuroepithelium in zebrafish. Thirdly, stem cell differentiation and adherence may be affected by defective basement membrane. We observed that the retinal development and RPC differentiation were delayed in USH2A mutation organoids at days 0–18 compared to healthy organoids, and the specific manifestation was that the RPC makers *PAX6*, *RAX*, *SIX6*, and *CHX10* were downregulated in the thinned retinal neuroepithelium. Serjanov et al. (2018) showed that deletion of *Lamb2* exhibited a loss of RPC basal processes that lead to decreased RPC proliferation and altered cellular composition of the retina in mice. Gopalan et al. showed that RPCs adhered to the inner limiting membrane, which consists of laminins, such as α1, α5, β1, β2, γ1, γ2, and γ3, during the development of the retina (Varshney et al., 2015).

## Conclusion

By combining iPSCs and 3D retinal organoids technologies with panel sequencing, we were able to demonstrate the two disease-causing mutations in a patient with non-syndromic USH2A-associated RP were associated with abnormal NR and RPE development. We identified a novel pathogenic mutation (c.8559-2A > G/c.9127_9129delTCC) in USH2A that has not been reported previously. Furthermore, we revealed that UCs cultured from a patient can be reprogrammed into iPSCs and further form a multilayered 3D retina organoid structure. Moreover, the resulting 3D retinas in the early stages display abnormal structure and function, including reduced organoid diameter and thickness, reduced laminin, increased apoptosis, and dysregulated RPC gene expression, defective photoreceptors and RPE cell phenotype. Abnormal retinal organoids with USH2A mutant correspond to RP disease characteristics such as atrophic pigment mottling on the fundus, decreased neuroepithelial and ONLs from OCT images, and reduced ERG a-wave and b-wave amplitude.

The retinal organoids enable to recapitulate human retinal development and disease that are not easily, early and accurately modeled in animals (Li and Izpisua Belmonte, 2019). Such kind of patients can immensely benefit, in terms of early prophylaxis, from the early pinpointing molecular diagnosis. The greatest benefit of the study genotype – phenotype – organoids correlations will allow the early molecular diagnosis as well as target based pathogenic mechanisms and intervention treatment. Being able to analyze and predict the course of the disease early in the process is highly desirable and becomes paramount (Pierrache et al., 2016; Jouret et al., 2019). Although iPS derived organoids can provide a unique platform for understanding diseases, it is important to keep in mind that the current organoids are very simplistic and immature (Qian et al., 2019). Organoids are semi-physiologic models because they are no vascularization and immune system (Li and Belmonte, 2019). Specially, the human retinal organoids require longer time to matured development (Lancaster and Knoblich, 2014). Our study is the first to investigate the effect of USH2A gene mutation on RP using 3D retinal organoid technology based on the related literature search. The limitation of this study is to involve early development in USH2A-associated organoids. Long-term culture of retinal organoid may recapitulate the phenotype more adequately. Further development of technologies, such as accelerating functional maturation and incorporating glia, microglia and vessel cells, will push our study toward more comprehensive and faithful RP models. Therefore, to overcome the technical challenges retinal organoid culture with USH2A mutation, for example easily disintegrated organoids, will be our future efforts. Additionally, our study just relies on comparison of one patient derived iPSC lined versus one healthy control. Next, we will screen more USH2A mutant RP cases including the proband pedigree by sequencing. Furthermore, applying patient derived iPSC-organoids system after CRISPR/Cas9-mediated correction of the mutation in treatment of RP is attractive, and the replacement of retinal tissue may inspire potential therapies (Jin et al., 2018).

## Data Availability

The data that support the findings of this study are available from the corresponding author upon reasonable request.

## Ethics Statement

This study was approved by the ethical committee of Aier Eye Institution and adhered to the tenets of the Declaration of Helsinki.

## Author Contributions

JC and YG did the background research and study design. JM collected the clinical data. ZC, DZ, YX, and QY analyzed and interpreted the data. SL, JC, and PW carried out the immunostaining analysis. YG drafted the manuscript. ZL and JC critically reviewed and revised the manuscript for intellectual content. JC and ST supervised the study and provided mentorship. All authors contributed to the important intellectual content during manuscript drafting or revision, and accepted the accountability for the overall work.

## Conflict of Interest Statement

The authors declare that the research was conducted in the absence of any commercial or financial relationships that could be construed as a potential conflict of interest.

## Footnotes

1 https://sph.uth.edu/retnet/sum-dis.htm

2 USHbases; http://www.lovd.nl/USH2A

3 http://www.mutationtaster.org/
