# Supplementary material for: Modeling Retinitis Pigmentosa: Retinal Organoids Generated From the iPSCs of a Patient With the USH2A Mutation Show Early Developmental Abnormalities
Source: Front Cell Neurosci. 2019 Aug 7;13:361. doi: 10.3389/fncel.2019.00361 (PMC6709881; doi:10.3389/fncel.2019.00361)
Supplement: Supplementary file 1 [file Data_Sheet_1.docx]

**Supplemental Information**

**Modeling retinitis pigmentosa: retinal organoids generated from iPSCs of a patient with USH2A mutation show developmental abnormalities**

Yonglong Guo, Peiyuan Wang, Jacey Hongjie Ma, Zekai Cui, Quan Yu, Shiwei Liu, Yunxia Xue, Deliang Zhu, Jixing Cao, Zhijie Li, Shibo Tang, Jiansu Chen

**Extended Experimental materials and Methods**

**Materials**

| **Name** | **Cat. No.** | **Company** |
| --- | --- | --- |
| CytoTune™-iPS 2.0 Sendai Reprogramming Kit | A16518 | ThermoFisher |
| Basic fbroblast growth factor (bFGF) | PHG0261 | ThermoFisher |
| BMP4 Recombinant Human Protein | PHC9534 | ThermoFisher |
| CTS™ (Cell Therapy Systems) N-2 Supplement | A13707-01 | ThermoFisher |
| Trypsin-EDTA (0.25%), phenol red (Gibco™) | 25200-056 | ThermoFisher |
| Essential 8 Medium (Gibco™) | A15169-01 | ThermoFisher |
| Essential 6™ Medium (Gibco™) | A15165-01 | ThermoFisher |
| DPBS, no calcium, no magnesium | 14190-136 | ThermoFisher |
| 0.5M EDTA PH 8.0 | 00552620 | ThermoFisher |
| Knockout Serum Replacement | 11710-035 | ThermoFisher |
| Chemically defined lipid concentrate | 11905031 | ThermoFisher |
| Iscove's Modified Dulbecco's Medium (IMDM) | 12440-053 | ThermoFisher |
| Ham's F-12 Nutrient Mix, GlutaMAX™ | 31765-035 | ThermoFisher |
| DMEM/F-12, GlutaMAX™ | 10565-018 | ThermoFisher |
| DMEM/F12 | 11320082 | ThermoFisher |
| Sodium Pyruvate 100 mM Solution | 11360-070 | ThermoFisher |
| MEM Non-Essential Amino Acids Solution | 11140-050 | ThermoFisher |
| Chemically Defined Lipid Concentrate | 11905-031 | ThermoFisher |
| Urineasy urine cell isolation kit | CA3102500 | Cellapy technology |
| Urineasy urine cell proliferation kit | CA3103200 | Cellapy technology |
| monothioglycerol | T109442 | Aladdin |
| Y-27632 | HY-10071 | MedChemExpress |
| Retinoic acid | HY-14649 | MedChemExpress |
| Taurine | HY-B0351 | MedChemExpress |
| CHI99021 | HY-10182 | MedChemExpress |
| SU5402 | HY-10407 | MedChemExpress |
| PrimeSurface MS-9096V | MS-9096V | Sumitomo Bakelite Co., Ltd. |

**Methods**

**Retinal organoid differentiation from human iPSCs**

Retinal organoid was generated from human iPSCs using a method as previously reported with some modifications [1]. Human iPSCs were digested into single cells and reseeded in low-cell-adhesion 96-well plates with V-bottomed conical wells at a density of 12,000 cells per well in NR induction medium supplemented with 20 μM Y-27632 under 5% CO_2_ conditions. On day 6, recombinant human BMP4 was added into a culture well at a concentration of 1.5 nM, and its concentration was diluted into half by half medium change every third day. On day 18, the NR containing aggregates were transferred onto low-cell-adhesion 6-well plates (6 aggregates per well) in RPE-induction medium supplement with CHI99021 (3μM) and SU5402 (5μM) under 5% CO_2_ conditions for 6 days culture. On day 24, the aggregates with RPE-like were cultured in NR and RPE induction medium under 40%O2/5%CO2 conditions for 6 days culture for long term culture.

NR induction medium containing 10% KSR, 45% Iscove’s modified Dulbecco’s medium, 45% Hams F12, Glutamax, 450 μM monothioglycerol, 1% chemically defined lipid concentrate, 100 U ml-1 penicillin and 100 μg ml-1 streptomycin. RPE-induction medium containing DMEM/F12-Glutamax, 1% N2 supplement, 3 mM, 100 U ml-1 penicillin and 100 μg ml-1 streptomycin. NR and RPE induction medium containing DMEM/F12-Glutamax, 1% N2 supplement, 10% FBS, 0.5 mM retinoic acid, 0.1 mM taurine, 0.25 μg/ml Fungizone, 100 U ml-1 penicillin and 100 μg ml-1 streptomycin.

**RPE cells differentiation**

RPE cells were differentiated from 3D retinal organoids following the method previously published with some modifications [2]. On day 34, identified pigmented patches were cut around from retinal organoid aggregate and cultured onto 6-well plates coated with 0.1% gelatin with RPE medium containing DMEM/F12, 1% MEM nonessential amino acids, 1% N2 supplement, 100 U ml-1 penicillin and 100 μg ml-1 streptomycin. The medium was changed every 2-3 day.

**Supplement figures and tables**


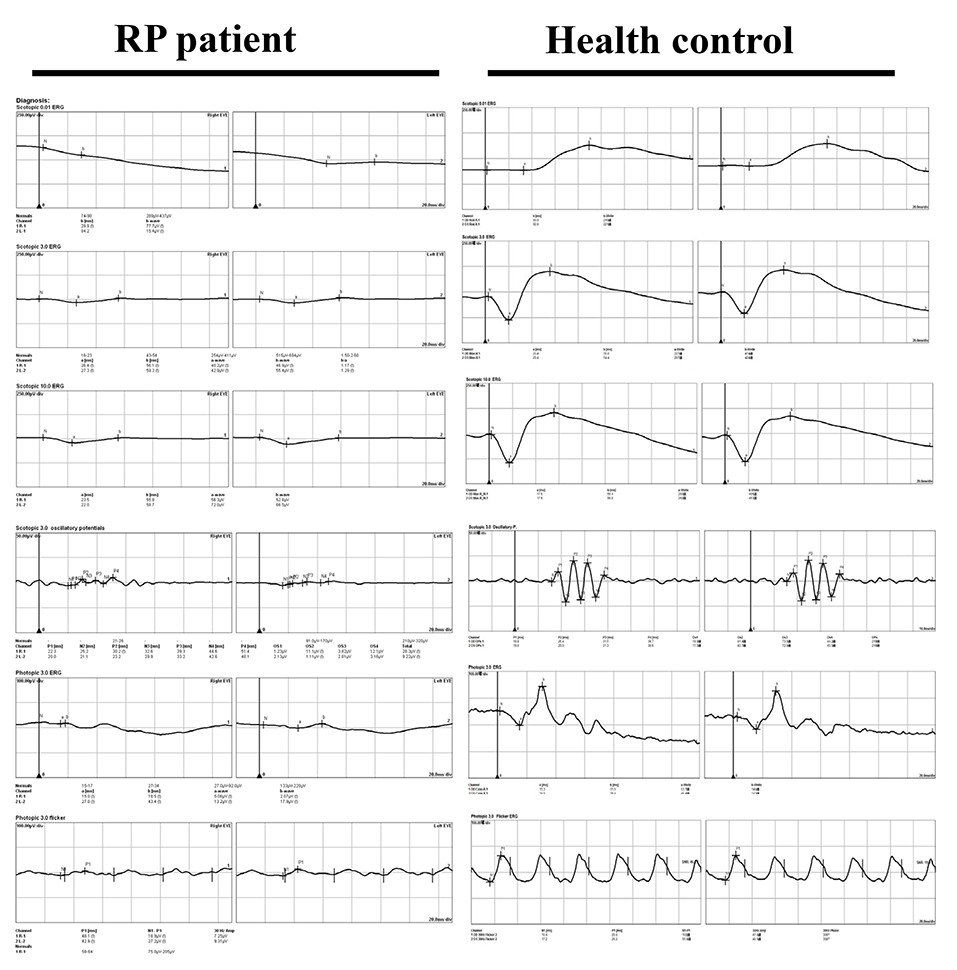


**Figure S-1**: Distinguished rod amplitude of Full-field ERG was detected in the proband (left panel), and the ff-ERG from health control were showed in the right panel.


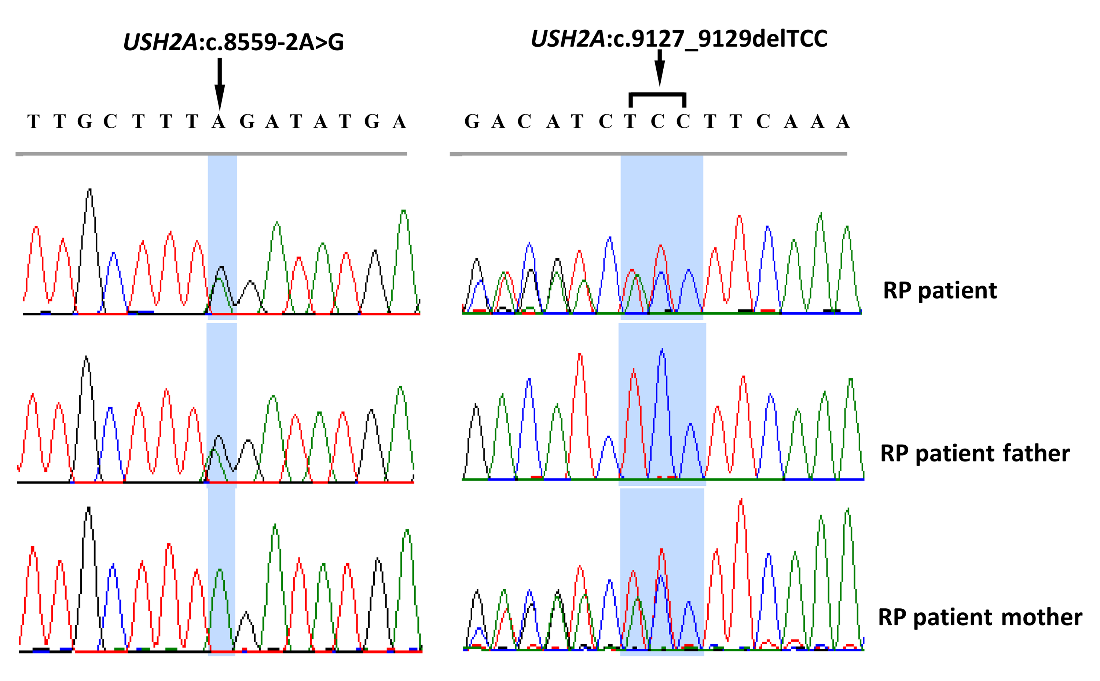


**Figure S-2**: The patient’ parental verification showed that father carries (USH2A; c.8559-2A>G) mutation and mother carries (c.9127_9129delTCC) mutation.

**A**


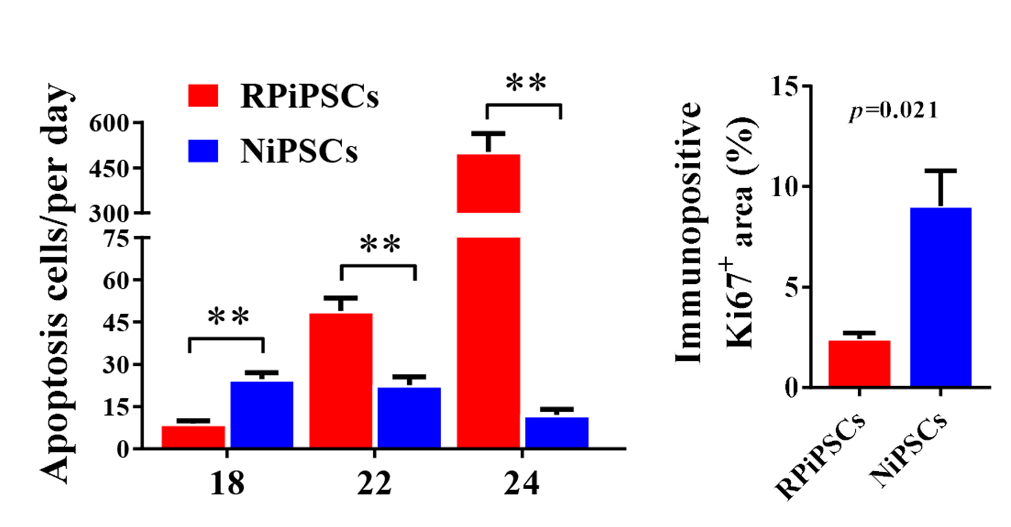

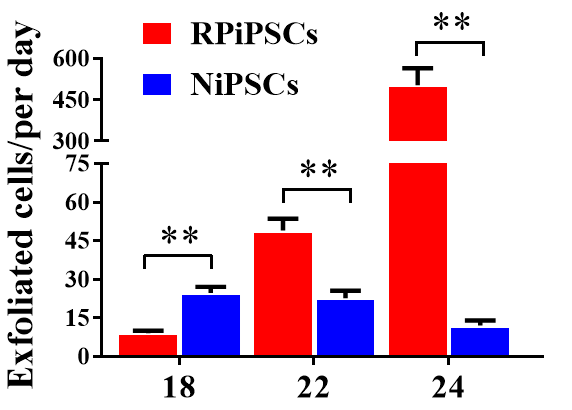


**B**

**Figure S-3**: (A) An interleaved bars graph shows the exfoliated cells of organoids per day in day 18, 22 and 24. (B) A separated bars graph shows the percentage of Ki67+ area in NR organoid expressing Ki67 immunoreactivities. (n=3 independent experiments; each experiment need 2- 4 organoids)


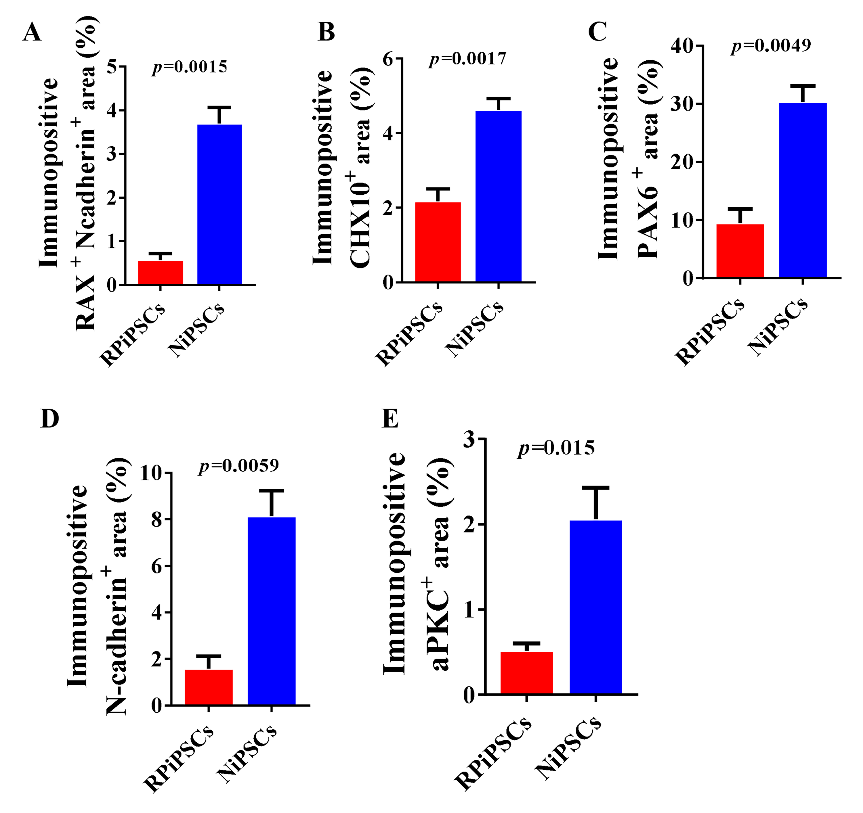


**Figure S-4**: A separated bars graph shows the percentage of RAX^+^ N-cadherin^+^(A), CHX10^+^ (B), PAX6^+^ (C), Ncadherin^+^ (D), aPKC^+^ (E) area in organoids immunostaining. (n=3 independent experiments; each experiment need 2- 4 organoids)


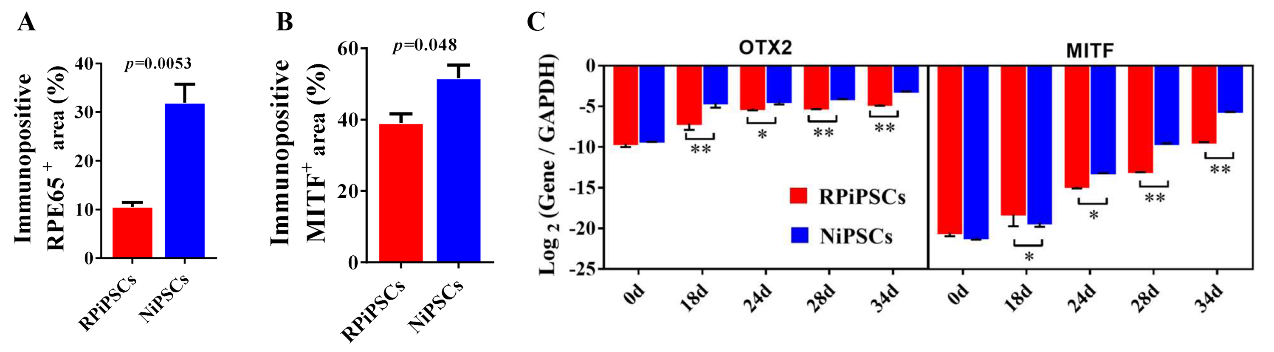


**Figure S-5**: A separated bars graph shows the percentage of RPE65^+^ (A), MITF^+^ (B) area in NR-RPE organoids expressing RPE65 and MITF immunostaining. (C) qPCR analysis revealed mRNA levels of transcripts corresponding to RPE functional process regulators (GAPDH gene as a control). (n=3 independent experiments; each experiment need 2- 4 organoids)


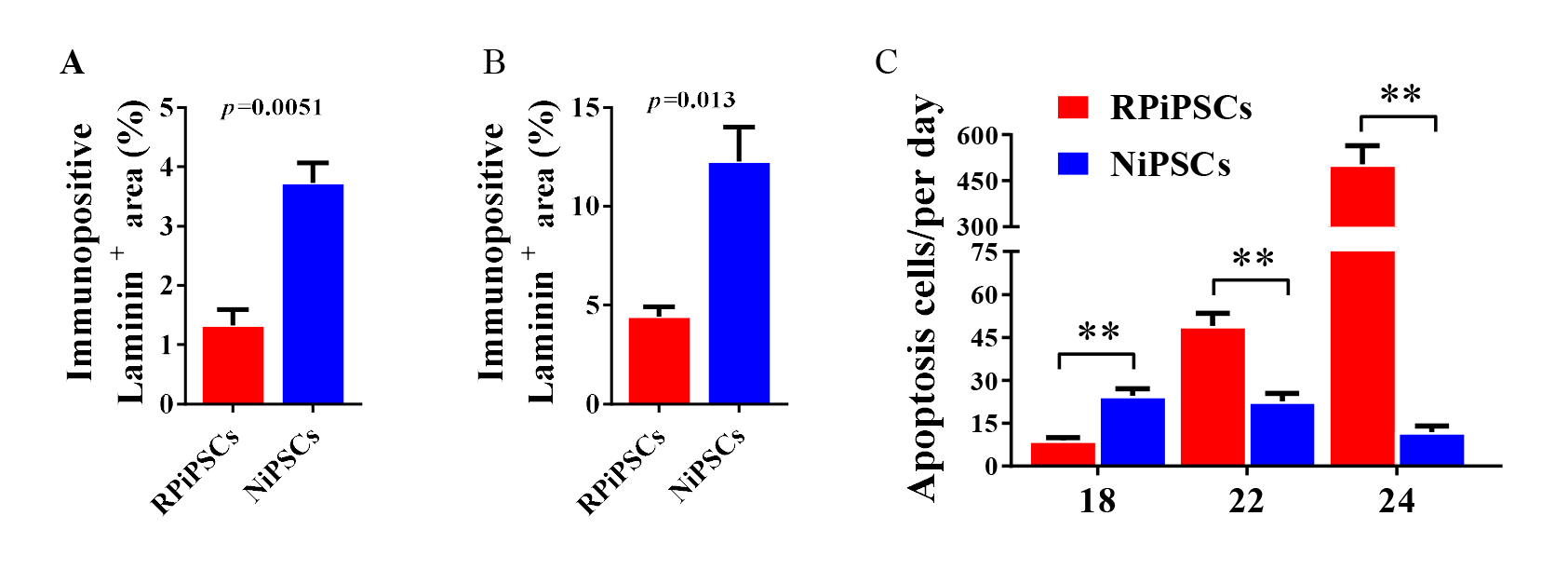


**Figure S-6**: A separated bars graph shows the percentage of Laminin^+^ area in organoids expressing Laminin immunostaining at day 18 (A) and day 26 (B). (n=3 independent experiments; each experiment need 2- 4 organoids)


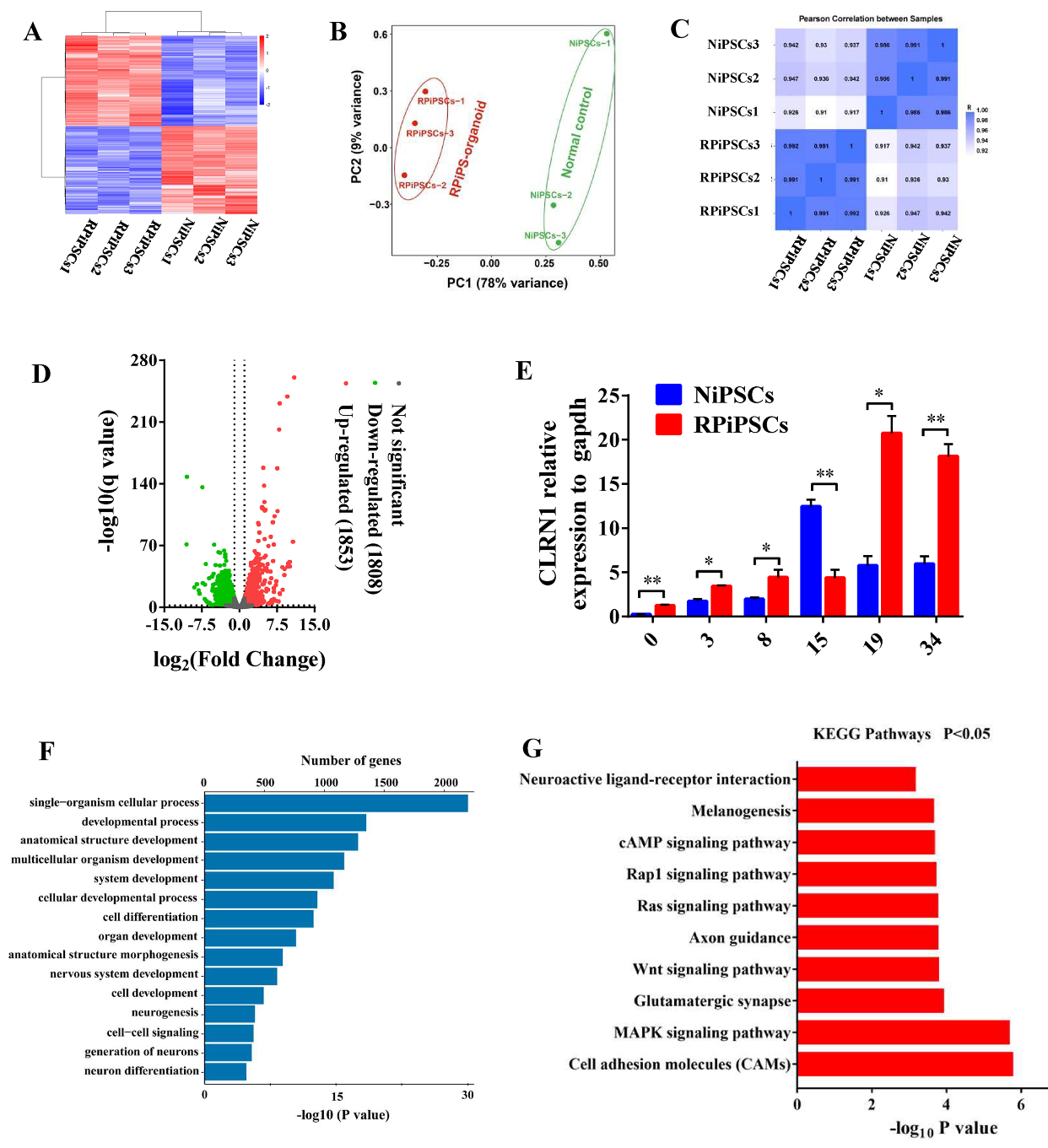


**Figure S-7**: Transcriptome data showed significant differences between the control and RP organoids. (A) The cluster heatmap of dataset. (B) The PCA showed that significant differences between the two groups of samples. (C) Pearson heatmap showed the correlation between the two groups of samples. (D) The volcano plot showed the distribution of all genes by fold change and P value. Genes with | log2 (fold change) | > 1 and P value < 0.05 were selected as the DEGs. There were 1853 DEGs up-regulated and 1808 DEGs were down-regulated. Red dots represent up-regulated genes, grey dots represent no significant genes, and green dots represent down-regulated genes, Bar is represents Z-score. GO enrichment for term analysis (F) and KEGG pathways (G) of the differentially expressed genes. (GAPDH gene as a control)

**Reference**

1. Kuwahara, A., et al., *Generation of a ciliary margin-like stem cell niche from self-organizing human retinal tissue.* Nature communications, 2015. **6**: p. 6286.

2. Reichman, S., et al., *Generation of Storable Retinal Organoids and Retinal Pigmented Epithelium from Adherent Human iPS Cells in Xeno‐Free and Feeder‐Free Conditions.* Stem Cells, 2017. **35**(5): p. 1176-1188.
